# Pharmacological study of the mechanisms involved in the vasodilator effect produced by the acute application of triiodothyronine to rat aortic rings

**DOI:** 10.1590/1414-431X20165304

**Published:** 2016-07-25

**Authors:** J. Lozano-Cuenca, O.A. López-Canales, J.C. Aguilar-Carrasco, J.R. Villagrana-Zesati, R.M. López-Mayorga, E.F. Castillo-Henkel, J.S. López-Canales

**Affiliations:** 1 Department of Cellular Biology, National Institute of Perinatology, Mexico City, Mexico; 2 Section of Postgraduate Studies and Investigation, Higher School of Medicine, National Polytechnic Institute, Mexico City, Mexico; 3 Department of Infectology and Perinatal Immunology, National Institute of Perinatology, Mexico City, Mexico

**Keywords:** Triiodothyronine, Rat aorta, Vasorelaxation, NO-cGMP/PKG pathway, K^+^ channels

## Abstract

A relationship between thyroid hormones and the cardiovascular system has been well established in the literature. The present *in vitro* study aimed to investigate the mechanisms involved in the vasodilator effect produced by the acute application of 10^-8^–10^-4^ M triiodothyronine (T_3_) to isolated rat aortic rings. Thoracic aortic rings from 80 adult male Wistar rats were isolated and mounted in tissue chambers filled with Krebs-Henseleit bicarbonate buffer in order to analyze the influence of endothelial tissue, inhibitors and blockers on the vascular effect produced by T_3_. T_3_ induced a vasorelaxant response in phenylephrine-precontracted rat aortic rings at higher concentrations (10^-4.5^–10^-4.0^ M). This outcome was unaffected by 3.1×10^-7^ M glibenclamide, 10^-3^ M 4-aminopyridine (4-AP), 10^-5^ M indomethacin, or 10^-5^ M cycloheximide. Contrarily, vasorelaxant responses to T_3_ were significantly (P<0.05) attenuated by endothelium removal or the application of 10^-6^ M atropine, 10^-5^ M L-NG-nitroarginine methyl ester (L-NAME), 10^-7^ M 1H-(1,2,4)oxadiazolo[4,3-a]quinoxalin-1-one (ODQ), 10^-6^ M (9*S*,10*R*,12*R*)-2,3,9,10,11,12-Hexahydro-10-methoxy-2,9-dimethyl-1-oxo-9,12-epoxy-1*H*-diindolo[1,2,3-*fg*:3′,2′,1′-*kl*]pyrrolo[3,4-*i*](1,6)benzodiazocine-10-carboxylic acid, methyl ester KT 5823, 10^-2^ M tetraethylammonium (TEA), or 10^-7^ M apamin plus 10^-7^ M charybdotoxin. The results suggest the involvement of endothelial mechanisms in the vasodilator effect produced by the acute *in vitro* application of T_3_ to rat aortic rings. Possible mechanisms include the stimulation of muscarinic receptors, activation of the NO-cGMP-PKG pathway, and opening of Ca^2+^-activated K^+^ channels.

## Introduction

Numerous experimental and clinical studies have demonstrated a relationship between thyroid hormones and the cardiovascular system, including reports of significant changes in cardiac function in patients with persistent subclinical thyroid dysfunction (1–5). Triiodothyronine (T_3_) and thyroxine (T_4_) are thyroid hormones present in plasma and peripheral tissues (6). T_3_ is mostly generated by 5′-monodeiodination of T_4_ in peripheral tissues (6,7). The acute application of T_3_ has been linked to a vasorelaxant effect (3,8–12), which has both an endothelium-independent and endothelium-dependent component. The endothelium-independent effect predominates in physiological concentrations and the endothelium-dependent effect in supraphysiological concentrations (13). The vasorelaxant endothelium-dependent effect produced by T_3_ has been linked to the activation of the endothelial nitric oxide synthase (eNOS), via thyroid hormone receptor/phosphatidylinositol 3-kinase/protein kinase-B pathway (TR/PI3-kinase/Akt pathway) (14). However, further research is needed about the possible involvement of muscarinic receptors, the nitric oxide-cyclic guanosine monophosphate-protein kinase G pathway (NO-cGMP-PKG pathway), and K^+^ channels in this vasorelaxant effect.

The present study aimed to analyze the effect of endothelium removal as well as the application of atropine, L-NG-nitroarginine methyl ester (L-NAME), 1H-(1,2,4)oxadiazolo[4,3-a]quinoxalin-1-one (ODQ), KT 5823, glibenclamide, 4-aminopyridine (4-AP), tetraethylammonium (TEA), apamin plus charybdotoxin, indomethacin and cycloheximide on the vasorelaxant response produced by supraphysiological concentrations (10^-8^–10^-4^ M) of T_3_ in phenylephrine-precontracted rat aortic rings.

## Material and Methods

### Animals

Experiments were performed on isolated thoracic aortic rings of adult male Wistar rats (body weight 250-300 g). Rats (n=80) were purchased from the bioterium of the Higher School of Medicine in the National Polytechnic Institute (Mexico City, Mexico). Animals were housed in plastic cages in a special temperature-controlled room (22±2°C, 50% humidity) on a 12:12 h light/dark cycle (lights on at 7:00 a.m.). The study was approved by the Animal Care Committee of the Higher School of Medicine and the protocol is in agreement with the 1986 Animals (Scientific Procedures) Act of the British Parliament (http://www.legislation.gov.uk/ukpga/1986/14/contents, accessed April 5, 2016).

### Preparation of aortic rings

Animals were euthanized by decapitation and the aortas were immediately excised and placed in cold buffer, cleaned and freed from surrounding connective tissue. The isolated arteries were cut into rings (4–5 mm long) and placed in 10 mL tissue chambers filled with Krebs-Henseleit bicarbonate buffer (1.18×10^-1^ M NaCl; 4.7×10^-3^ M KCl; 1.2×10^-3^ M KH_2_ PO_4_; 1.2×10^-3^ M MgSO_4_.7H_2_O; 2.5 × 10^-3^ M CaCl_2_·2H_2_O; 2.5×10^-2^ M NaHCO_3_; 1.17×10^-2^ M dextrose, and 2.6×10^-5^ M calcium disodium EDTA). In some experiments, the KCl concentration was increased to 8×10^-2^ M and that of Na^+^ decreased to maintain osmotic equilibrium. Tissue baths, maintained at 37°C and pH 7.4, were bubbled with a mixture of 95% O_2_ and 5% CO_2_.

Aortic rings were mounted on two stainless steel hooks, one fixed to the bottom of the chamber and the other to a BIOPAC TSD125C-50g force transducer connected to a BIOPAC MP100A-CE data acquisition system (Biopac Systems, Inc., USA) in order to record the isometric tension. Optimal tension, selected from preliminary experiments, was the one that gave the greatest response to 10^-6^ M phenylephrine. The rings were given 2 *g* (100%) of initial tension and allowed to equilibrate for 2 h. Thirty minutes after setting up the organ bath, tissues were contracted with 10^-6^ M phenylephrine to test their contractile responses.

Endothelium-denuded aortic strips were prepared by turning the rings gently several times on the distal portion of small forceps. Endothelial integrity was pharmacologically assessed with acetylcholine-induced vasodilatation (10^-6^ M). Segments showing no relaxation to acetylcholine were considered to be endothelium-denuded. After exposure to 10^-6^ M phenylephrine or 10^-6^ M acetylcholine, tissues were rinsed three times with Krebs solution to restore basal tension.

### Drugs

All drugs except T3 were purchased from Sigma-Aldrich Co. (USA). T_3_ was a gift from Productos Medix, S.A. de C.V (Mexico). Atropine, L-NAME, 4-AP, TEA and cycloheximide were dissolved in distilled water. Solutions of 10^-5^ M ODQ, 10^-4^ M KT 5823, 10^-5^ M apamin plus 10^-5^ M charybdotoxin and 10^-3^ M indomethacin were prepared by using 1.39 M dimethyl sulfoxide, 1.01 M ethyl acetate, 1.73 M acetic acid and 9.4×10^-3^ M sodium bicarbonate, respectively. Solutions of T_3_ were prepared with serial dilutions as follows: a stock solution of 10^-2^ M T_3_ was made in an aqueous solution of 2.5 M NaOH, and subsequently dilutions of 10^-2.5^, 10^-3^, 10^-3.5^, 10^-4^, 10^-4.5^, 10^-5^, 10^-5.5^ and 10^-6^ M T_3_ were prepared in 1.38, 1.38×10^-1^, 1.38×10^-2^, 1.38×10^-2^, 1.38×10^-3^, 1.38×10^-3,^ 1.38×10^-4^ and 1.38×10^-4^ M NaOH, respectively. Fresh solutions were made for each experiment.

### Experimental protocol

To determine the mechanisms involved in the relaxant effect induced by T_3_ on phenylephrine-precontracted rat aortic rings, two main sets of experiments were performed.

*First set of experiments*. Thirty minutes after restoration of basal tension (see Preparation of aortic rings section), 10^-6^ M phenylephrine was added to rat aortic rings with or without endothelium. Twenty minutes later, the phenylephrine-induced contraction plateaued. Thirty minutes after adding phenylephrine, T_3_ began to be cumulatively added (10^-8^–10^-4^ M) in intervals of around 4 min. Tension is reported as a percentage of the phenylephrine-induced contraction (3.79±0.16 g = 100% for endothelium-intact rat aortic rings and 4.21±0.13 g = 100% for endothelium-denuded rings). With cumulative addition into the tissue chambers, dilutions of T_3_ (prepared in aqueous solutions of NaOH; see Drugs section) reached concentrations of 2.5×10^-2^, 1.38×10^-2^, 1.38×10^-3^, 1.38×10^-3^, 1.38×10^-4^, 1.38×10^-4^, 1.38×10^-5^, 1.38×10^-5^, 1.38×10^-6^ and 1.38×10^-6^ M NaOH.

*Second set of experiments*. Thirty min after adding 10^-6^ M phenylephrine (see first set of experiments), rat aortic rings with intact endothelium were preincubated for 30 min with one of the inhibitors, blockers or vehicles: i) 10^-6^ M atropine, ii) 10^-5^ M L-NAME, iii) 10^-7^ M ODQ, iv) 10^-6^ M KT 5823, v) 3.1×10^-7^ M glibenclamide, vi) 10^-3^ M 4-AP, vii) 10^-2^ M TEA, viii) 10^-7^ M apamin plus 10^-7^ M charybdotoxin, ix) 10^-5^ M indomethacin, x) 10^-5^ M cycloheximide, xi) distilled water (vehicle of atropine, L-NAME, 4-AP, TEA, and cycloheximide), xii) 1.39×10^-2^ M dimethyl sulfoxide (vehicle of ODQ), xiii) 1.01×10^-2^ M ethyl acetate (vehicle of KT 5823), xiv) 1.73×10^-2^ M acetic acid (vehicle of apamin plus charybdotoxin), or xv) 9.4×10^-5^ M sodium bicarbonate (vehicle of indomethacin). Subsequently, T_3_ was cumulatively added in approximately 4-min intervals to reach a concentration between 10^-8^ and 10^-4^ M. Once reaching the desired concentration, the vasorelaxant response of the rings was assessed.

### Data analysis and statistics

Data are reported as means±SE. In all experiments, n equals the number of animals from which aortic segments were obtained (8 in each case). Values of maximal vasorelaxation (E_max_) were analyzed by Student's *t*-test. Effects of inhibitors/blockers on the vasorelaxant responses produced by T_3_ on phenylephrine-precontracted aortic segments were analyzed by a two-way analysis of variance (ANOVA), which was followed by a Student-Newman-Keul's *post hoc* test. Statistical significance was considered at P<0.05 (15). Statistical analyses were performed with the SigmaPlot 12 program (Systat Software Inc., USA).

## Results

### Effect of T3 on endothelium-intact and -denuded phenylephrine-precontracted rat aortic rings

**Figure 1:**
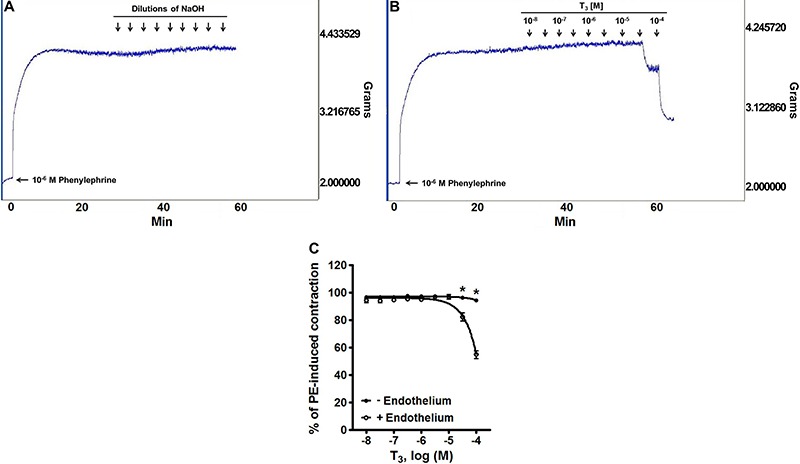
Original experimental tracings illustrating the *in vitro* effect in phenylephrine (PE)-precontracted rat aortic rings produced by the application of: *A*, dilutions of NaOH (vehicle of T_3_), *B*, 10^-8^–10^-4^ M T_3_. A T_3_-induced vasorelaxant response can be seen at the highest concentrations of this hormone. Similar results were obtained in all assays (n=8). Endothelial denudation blocked the vasorelaxation produced by higher concentrations of T_3_ in PE-precontracted rat aortic rings (*C*). Data are reported as means±SE (n=8 for each group). *P<0.05 *vs* control (two-way ANOVA).

Figure 1A and B shows typical traces of the effect produced by the *in vitro* application of dilutions of NaOH (vehicle of T_3_) and 10^-8^–10^-4^ M T_3_ on phenylephrine-precontracted rat aortic rings with intact endothelium. The addition of 10^-6^ M phenylephrine to rat aortic rings produced a sustained contraction. The cumulative addition of T_3_ (10^-8^–10^-4^ M) produced a concentration-dependent vasorelaxant response, which was not observed with the vehicle (dilutions of NaOH). Figure 1C shows the effect of the cumulative addition of 10^-8^–10^-4^ M T_3_ to phenylephrine-precontracted rat aortic rings. When comparing endothelium-intact and -denuded rings, the E_max_ was 45.09±2.77 *vs* 5.44±0.97%, respectively, representing a significant difference (P<0.05).

### Effect of atropine on the vasorelaxation induced by T3 in phenylephrine-precontracted rat aortic rings

**Figure 2:**
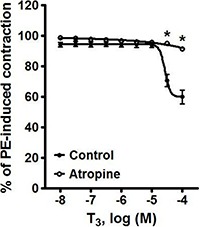
Pre-incubation with 10^-6^ M atropine blocked the vasorelaxation produced by higher concentrations of T_3_ in phenylephrine (PE)-precontracted rat aortic rings. Data are reported as means±SE (n=8). *P<0.05 *vs* control (two-way ANOVA).

Figure 2 shows the effect of 10^-6^ M atropine on the vasorelaxation induced by 10^-8^–10^-4^ M T_3_ in phenylephrine-precontracted rat aortic rings. When comparing the absence and presence of atropine, the values of E_max_ were 40.10±4.64 *vs* 8.51±1.07, respectively, representing a significant difference (P<0.05).

### Effect of L-NAME, ODQ and KT 5823 on the vasorelaxation induced by T3 in phenylephrine-precontracted rat aortic rings

**Figure 3:**
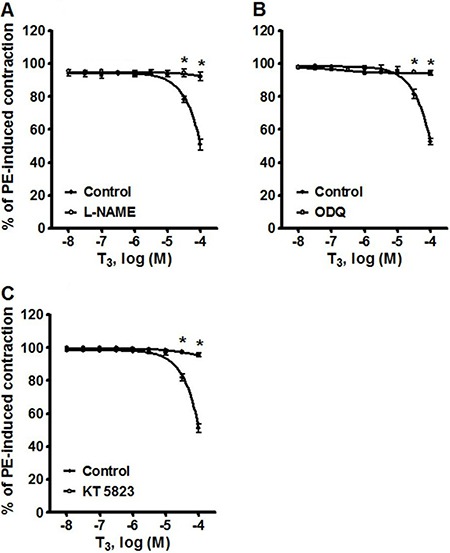
Vasorelaxation produced by 10^-8^–10^-4^ M T_3_ in phenylephrine (PE)-precontracted rat aortic rings. Assays were carried out to test the effect of: *A*, 10^-5^ M L-NG-nitroarginine methyl ester (L-NAME), *B*, 10^-7^ M 1H-(1,2,4)oxadiazolo[4,3-a]quinoxalin-1-one (ODQ), and *C,* 10^-6^ M (9*S*,10*R*,12*R*)-2,3,9,10,11,12-hexahydro-10-methoxy-2,9-dimethyl-1-oxo-9,12-epoxy-1*H*-diindolo[1,2,3-*fg*: 3′,2′,1′-*kl*]pyrrolo[3,4-*i*](1,6) benzodiazocine-10-carboxylic acid, methyl ester (KT 5823). Data are reported as means±SE (n=8). *P<0.05 *vs* control (two-way ANOVA).

Figure 3 shows the effect of 10^-5^ M L-NAME (A), 10^-7^ M ODQ (B) and 10^-6^ M KT 5823 (C) on the vasorelaxation induced by 10^-8^–10^-4^ M T_3_ in phenylephrine-precontracted rat aortic rings. The values of E_max_ from segments treated with T_3_ yielded a significant difference (P<0.05) when comparing the absence and presence, respectively, of each compound: 49.03±3.29 *vs* 7.74±2.62% for L-NAME, 47.42±1.74 *vs* 5.75±1.51% for ODQ, and 48.90±2.59 *vs* 4.43±1.33% for KT 5823.

### Effect of glibenclamide, 4-AP, TEA, and apamin plus charybdotoxin on the vasorelaxation induced by T3 in phenylephrine-precontracted rat aortic rings

**Figure 4:**
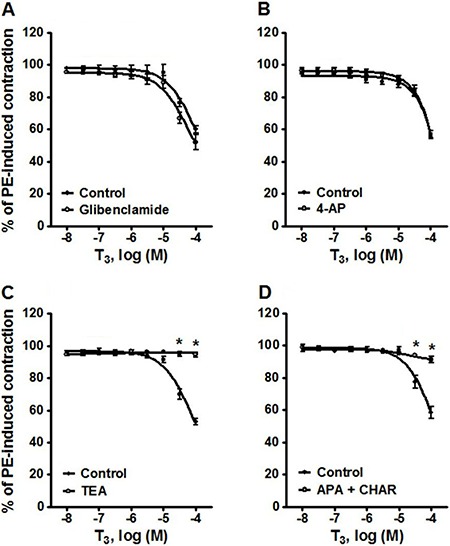
Vasorelaxation produced by 10^-8^–10^-4^ M T_3_ in phenylephrine (PE)-precontracted rat aortic rings. Assays were carried out to test the effect of: *A*, 3.1×10^-7^ M glibenclamide, *B*, 10^-3^ M 4-aminopyridine (4-AP), *C*, 10^-2^ M tetraethylammonium (TEA), and *D*, 10^-7^ M apamin plus 10^-7^ M charybdotoxin (APA+CHAR). Data are reported as means±SE (n=8). *P<0.05 *vs* control (two-way ANOVA).

Figure 4 shows the effect of 3.1×10^-7^ M glibenclamide (A), 10^-3^ M 4-AP (B), 10^-2^ M TEA (C), and 10^-7^ M apamin plus 10^-7^ M charybdotoxin (D) on the vasorelaxation induced by 10^-8^–10^-4^ M T_3_ in phenylephrine-precontracted rat aortic rings. The values of E_max_ represented a significant difference (P<0.05) only when comparing the absence and presence, respectively, of the latter two compounds: 39.87±2.29 *vs* 47.97±4.54% for glibenclamide, 42.97±2.44 *vs* 43.20±2.65% for 4-AP, 47.11±2.12 *vs* 5.34±1.50% for TEA, and 41.44±3.82 *vs* 8.69±1.97% for apamin plus charybdotoxin.

### Effect of indomethacin and cycloheximide on the vasorelaxation induced by T3 in phenylephrine-precontracted rat aortic rings

**Figure 5:**
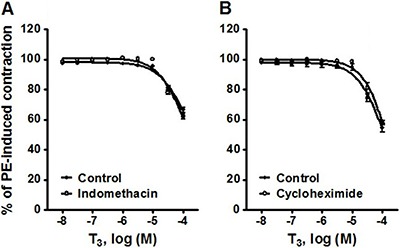
Vasorelaxation produced by 10^-8^–10^-4^ M T_3_ in phenylephrine (PE)-precontracted rat aortic rings. Assays were carried out to test the effect of: *A*, 10^-5^ M indomethacin, and *B*, 10^-5^ M cycloheximide. Data are reported as means±SE (n=8).

Figure 5 shows the effect of 10^-5^ M indomethacin (A) and 10^-5^ M cycloheximide (B) on the vasorelaxation induced by 10^-8^–10^-4^ M T_3_ in phenylephrine-precontracted rat aortic rings. The difference in the values of E_max_ when comparing the absence and presence, respectively, of each compound were not significant: 33.54±1.80 *vs* 37.77±1.85% for indomethacin and 45.44±2.88 *vs* 42.08±1.50% for cycloheximide.

### Effect of distilled water, dimethyl sulfoxide, ethyl acetate, acetic acid and sodium bicarbonate on the vasorelaxation induced by T3 in phenylephrine-precontracted rat aortic rings

**Figure 6:**
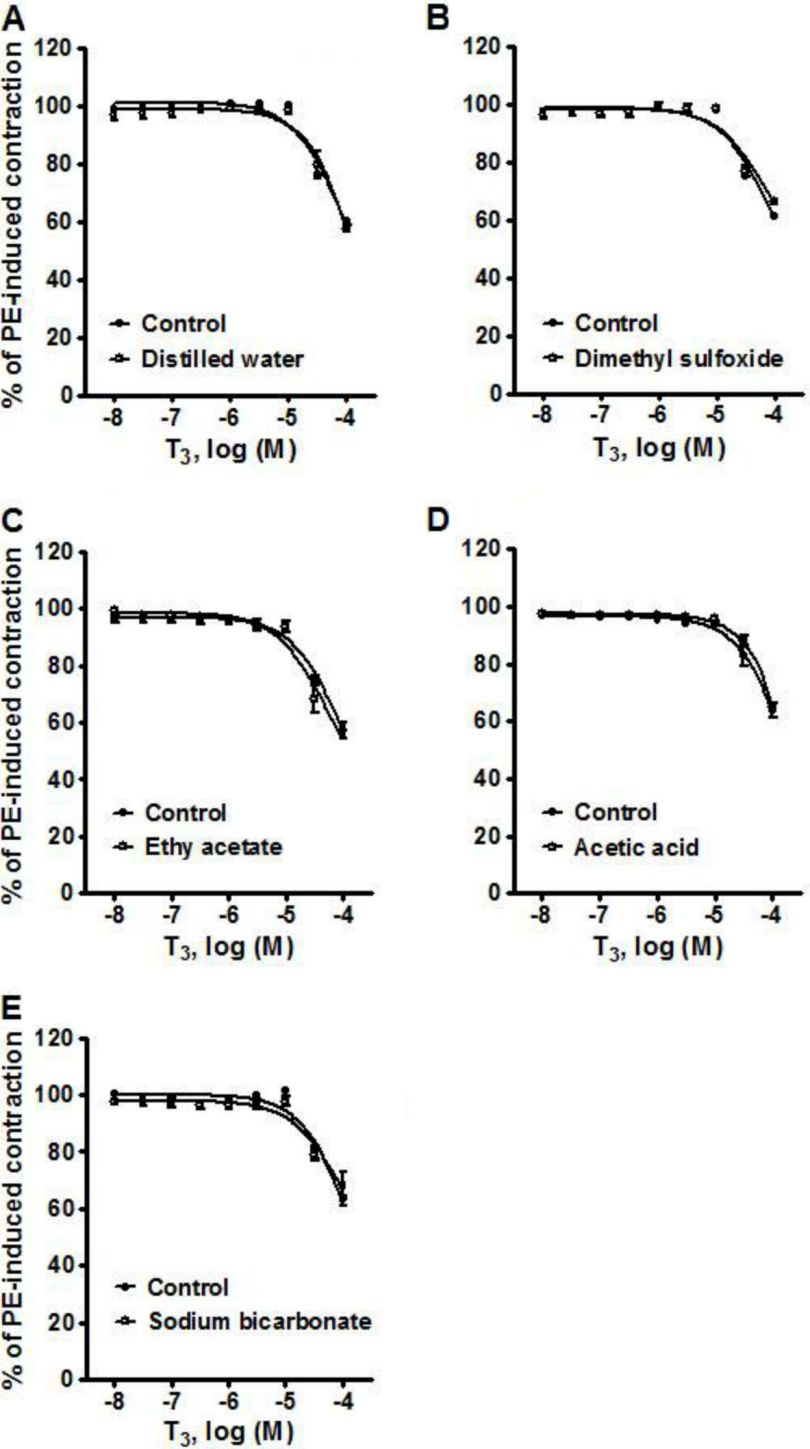
Vasorelaxation produced by 10^-8^–10^-4^ M T_3_ in phenylephrine (PE)-precontracted rat aortic rings. Assays were carried out to test the effect of: *A*: distilled water, *B*, 1.39×10^-2^ M dimethyl sulfoxide, *C*, 1.01×10^-2^ M ethyl acetate, *D*, 1.73×10^-2^ M acetic acid, and *E*, 9.4×10^-5^ M sodium bicarbonate. Data are reported as means±SE (n=8).

Figure 6 shows the effect on the vasorelaxation induced by 10^-8^–10^-4^ M T_3_ in phenylephrine-precontracted rat aortic rings produced by distilled water (vehicle of atropine, L-NAME, 4-AP, TEA and cycloheximide (A), 1.39×10^-2^ M dimethyl sulfoxide (vehicle of ODQ; B), 1.01×10^-2^ M ethyl acetate (vehicle of KT 5823; C), 1.73×10^-2^ M acetic acid (vehicle of apamin plus charybdotoxin; D), and 9.4×10^-5^ M sodium bicarbonate (vehicle of indomethacin; E). The difference in the values of E_max_ in the absence and presence, respectively, of each compound was not significant in any case: 39.56±1.27 *vs* 42.36±1.31% for distilled water, 38.73±0.48 *vs* 33.47±1.10% for dimethyl sulfoxide, 41.45±1.51 *vs* 44.12±1.60% for ethyl acetate, 36.31±2.58 *vs* 34.99±1.01% for acetic acid, and 37.20±1.89 *vs* 31.93±4.67% for sodium bicarbonate.

## Discussion

The acute (immediate) application of T_3_ produced an immediate vasorelaxant effect in endothelium-intact but not in endothelium-denuded phenylephrine-precontracted rat aortic rings, suggesting that this thyroid hormone produces an endothelium-dependent vasorelaxation. This vasorelaxant effect was statistically significant at higher concentrations of T_3_ (10^-4.5^–10^-4.0^ M), in line with previous findings in which the endothelium-dependent effect produced by T_3_ was most obvious in supraphysiological concentrations (13). However, our findings are in contrast with a previous report in segments of endothelium-denuded rat thoracic aorta, in which incubation for 30 min with 10^-7^ M T_3_ decreased the phenylephrine-induced contractile response (16). A possible explanation for this discrepancy could be that 10^-7^ M T_3_ cannot produce an immediate endothelium-dependent vasorelaxation and it is necessary to incubate for 30 min to see endothelium-independent vasorelaxant effects on the aortic tissues. Since the vehicle did not produce a concentration-dependent vasorelaxant effect in phenylephrine-precontracted rat aortic rings, it can be ruled out that the T_3_-induced vasorelaxation was due to tachyphylactic effects caused by the repeated application of dilutions of NaOH to aortic segments.

The vasorelaxant effect produced by 10^-8^–10^-4^ M T_3_ in phenylephrine-precontracted rat aortic rings is in agreement with numerous studies. It has been reported that: i) the bolus injection of T_3_ elicited an immediate and transient dose-dependent vasodilator effect in rat coronary arteries (11), ii) the acute application of 10^-8^–10^-4^ M L-T_3_ or 10^-8^–10^-4^ M D-T_3_ on rat mesenteric arteries produced a concentration-dependent vasorelaxant effect (10), iii) the acute application of 10^-10^–10^-7^ M T_3_ on rat skeletal muscle resistance arteries produced a concentration-dependent vasorelaxant effect (13), and iv) the acute application of 10^-6^–10^-4^ M L-T_3_ or 10^-6^–10^-4^ M D-T_3_ on rabbit mesenteric arteries produced a concentration-dependent vasorelaxant effect (8). However, the current results are in contrast with a previous study in which the cumulative application of 10^-7^–10^-4.5^ M T_3_ did not produce significant changes in rat aortic segments (3). Discrepancies in the reported vascular effect of T_3_ may be related to differences in experimental conditions, such as the concentrations of T_3_ applied and the type of vascular tissue studied (i.e., conductance or resistance vessels).

The current findings suggest that endothelium-independent mechanisms were not involved in this vasorelaxant effect. These findings contrast with two previous studies in which: after endothelial denudation, 10^-10^–10^-7^ M T_3_ produced a moderate vasorelaxant effect in rat skeletal muscle arteries (13), and the endothelial denudation of rat mesenteric and femoral arteries did not modify the vasorelaxant effect produced by 3×10^-7^-3×10^-5^ M T_3_ (3). These discrepancies could be due to: i) the time frame for the T_3_ stimulation of the endothelium-independent mechanisms (the application of all concentrations of T_3_ was herein performed in about 40 min, while previous studies took over 60 min for T_3_ application), and ii) the endothelium-independent mechanisms are more sensitive to T_3_ in resistance than in capacitance vessels.

An attempt was made to determine the endothelial mechanisms involved in the vasorelaxant effect found in endothelium-intact but not -denuded aortic rings. It is known that in the vasculature, the endothelial stimulation of muscarinic M_3_ and M_5_ receptors produces a vasorelaxant effect (17). The concentration of atropine employed herein (10^-6^ M), known to completely block muscarinic receptors (18), impeded the vasorelaxation produced by the acute application of supraphysiological concentrations of T_3_ in endothelium-intact aortic rings. However, distilled water (vehicle of atropine) did not impede such vasorelaxation (Figure 2), suggesting the possible involvement of muscarinic receptors. Further experiments, which fall beyond the scope of this investigation, are needed to identify the specific muscarinic receptor subtype(s) involved in the vasorelaxant effect produced by T_3_.

The current results also suggest the involvement of the NO-cGMP-PKG pathway in the vasorelaxant effect produced by 10^-8^–10^-4^ M T_3_ in rat aortic rings, since this effect was significantly attenuated by 10^-5^ M L-NAME (a direct inhibitor of NOS) (19), 10^-7^ M ODQ (an inhibitor of nitric oxide-sensitive guanylyl cyclase) (20), and 10^-6^ M KT 5823 (an inhibitor of protein kinase G) (21), but unaffected by the respective vehicles (distilled water), 1.39×10^-2^ M dimethyl sulfoxide) and 1.01×10^-2^ M ethyl acetate. These findings exclude the possibility that the attenuating effect produced by L-NAME, ODQ and KT 5823 were due to tachyphylactic effects induced by their respective vehicles.

On the other hand, 10^-7^ M ODQ and 10^-6^ M KT 5823, but not 10^-5^ M L-NAME inhibited the vasorelaxant responses to 10^-11^–10^-5^ M sodium nitroprusside (data not shown). These results suggest that the concentrations of ODQ and KT 5823 were high enough to inhibit the nitric oxide-sensitive guanylyl cyclase and the protein kinase G, respectively. Moreover, the fact that L-NAME did not modify the vasorelaxant effect to sodium nitroprusside suggests that this inhibitor acts specifically on the vasorelaxation dependent of the synthesis of nitric oxide.

The probable involvement of NO in the vasorelaxation produced by 10^-8^–10^-4^ M T_3_ in rat aortic rings is in line with previous studies suggesting that T_3_ exerts a direct effect on the regulation of vascular tone through non-genomic activation of eNOS, via the TR/PI3-kinase/Akt pathway (14). NO produced in endothelial cells by eNOS diffuses into vascular smooth muscle and directly activates soluble guanylate cyclase (22,23), leading to increased formation of cGMP. The resulting synthesis of cGMP is critical in mediating vasodilation through activation of PKG (24,25).

The present findings suggest the involvement of yet another mechanism – that of Ca^2+^-activated K^+^ channels – in the vasorelaxant response produced by 10^-8^–10^-4^ M T_3_ in phenylephrine-precontracted rat aortic rings. Vasorelaxation was unaffected by 3.1×10^-7^ M glibenclamide (an ATP-sensitive K^+^ channel blocker) (26) and 10^-3^ M 4-AP (a voltage-activated K^+^ channel blocker) (27,28), but was significantly (P<0.05) attenuated by 10^-2^ M TEA (a Ca^2+^-activated K^+^ channel blocker and non-specific voltage-activated K^+^ channel blocker) (27,29) and 10^-7^ M apamin plus 10^-7^ M charybdotoxin (blockers of small- and large-conductance Ca^2+^-activated K^+^ channels, respectively) (30–32). Moreover, the vasorelaxant response was unaffected by distilled water (vehicle of L-NAME, 4-AP and TEA) and acetic acid (vehicle of apamin plus charybdotoxin). This emphasizes the reproducibility of these results and rules out the possibility that attenuations produced by the K^+^ channel blockers are due to tachyphylactic effects.

The combination of apamin plus charybdotoxin was used because it was previously reported that a complete blockage of Ca^2+^-activated K^+^ channels is necessary to produce a pharmacological response (31–33). In this sense, a pilot experiment conducted in our laboratory showed that apamin alone did not modify the vasorelaxant response to 10^-8^–10^-4^ M T_3_ (data not shown). These observations suggest, but do not prove, that T_3_ produces vascular hyperpolarization attributable to the release of an endothelium-dependent hyperpolarizing factor. The above effect and mechanism was previously reported for acetylcholine (34,35). Certainly, this idea is still speculative and requires additional experiments that are beyond the scope of the present study.

There is a large body of evidence suggesting that prostacyclins (36) and protein synthesis (37) are involved in the endothelial control of vascular tone. However, the possible involvement of prostaglandin/protein synthesis in the vasorelaxation produced by T_3_ (38) is excluded by the current results in regard to indomethacin (a prostaglandin synthesis inhibitor) (39) and cycloheximide (a general protein synthesis inhibitor). Moreover, the lack of effect of cycloheximide on T_3_-induced vasorelaxation suggests that genomic mechanisms are not involved.

The present study showed that an acute *in vitro* application of supraphysiological concentrations of T_3_ in endothelium-intact rat aortic rings produced an immediate vasorelaxant effect. The *in vitro* character of this study represents a limitation. Although the current findings suggest an immediate vasorelaxant effect of T_3_, *in vivo* studies are needed to establish whether the administration of higher doses of T_3_ produces vasodepressor effects. Overall, the present results suggest some possible non-genomic mechanisms for the vasorelaxant effect observed – the NO-cGMP-PKG pathway and Ca^2+^-activated K^+^ channels via activation of muscarinic receptors.
